# Identification of a 12-Gene Signature and Hub Genes Involved in Kidney Wilms Tumor *via* Integrated Bioinformatics Analysis

**DOI:** 10.3389/fonc.2022.877796

**Published:** 2022-04-11

**Authors:** Guoping Huang, Jianhua Mao

**Affiliations:** Department of Nephrology, The Children’s Hospital, Zhejiang University School of Medicine, National Clinical Research Center for Child Health, Hangzhou, China

**Keywords:** miRNA, prognosis, Wilms tumor, target genes, risk model

## Abstract

Wilms tumor (WT), also known as nephroblastoma, is a rare primary malignancy in all kinds of tumor. With the development of second-generation sequencing, the discovery of new tumor markers and potential therapeutic targets has become easier. This study aimed to explore new WT prognostic biomarkers. In this study, WT-miRNA datasets GSE57370 and GSE73209 were selected for expression profiling to identify differentially expressed genes. The key gene miRNA, namely hsa-miR-30c-5p, was identified by overlapping, and the target gene of candidate hsa-miR-30c-5p was predicted using an online database. Furthermore, 384 genes were obtained by intersecting them with differentially expressed genes in the TARGET-WT database, and the genes were analyzed for pathway and functional enrichment. Kaplan–Meier survival analysis of the 384 genes yielded a total of 25 key genes associated with WT prognosis. Subsequently, a prediction model with 12 gene signatures (BCL6, CCNA1, CTHRC1, DGKD, EPB41L4B, ERRFI1, LRRC40, NCEH1, NEBL, PDSS1, ROR1, and RTKN2) was developed. The model had good predictive power for the WT prognosis at 1, 3, and 5 years (AUC: 0.684, 0.762, and 0.774). Finally, ERRFI1 (hazard ratios [HR] = 1.858, 95% confidence intervals [CI]: 1.298–2.660) and ROR1 (HR = 0.780, 95% CI: 0.609–0.998) were obtained as independent predictors of prognosis in WT patients by single, multifactorial Cox analysis.

## Introduction

Wilms tumor (WT), also known as nephroblastoma, is the second most common intra-abdominal tumor and the most common primary renal tumor in children (1). Approximately 75% of children with WT develop the condition between the ages of 1 and 5 years, most commonly at the age of (2). After years of clinical exploration, the five-year survival rate for WT has improved from less than 30% to 85%–90%. However, the recurrence rate remains at 15%–50% (3). The treatment and prognosis of nephroblastoma in children are related to histological staging (4), and five-year survival rates have reached 90% for children with good histological types of WT after moderate treatment (5, 6).

The treatment of nephroblastoma continues to be based on surgical resection supplemented by a combination of chemotherapy and radiotherapy (7, 8). However, treatment-related complications remain a problem for many children with WT, with treatment often triggering nausea and vomiting, loss of appetite, anemia, alopecia, and neutropenia, which subsequently affect patients’ psychological well-being (9). At the same time, surgical removal of the diseased kidney has limitations. Therefore, current treatments are not entirely appropriate for some populations, especially infants and children and patients with bilateral tumors (10). Therefore, the key to improving patient prognosis is to improve treatment based on clinical and biological risk factors, and further stratification of current treatment options based on the prognostic value of tumor biology would be an important approach to improving WT prognosis (11, 12). Along with the development of CRISPR/Cas9 gene-editing technology, artificially modified chimeric antigen receptor T cell immunotherapy, and aptamer technology, precise genetic and biological therapies could be a new option for the treatment of nephroblastoma (13–15).

With the development of second-generation sequencing, the discovery of new tumor markers and potential therapeutic targets has become more accessible. Advances in RNA sequencing technologies have revealed the complexity of the human genome. The study of the RNA transcriptome is one of the most important challenges facing biology today, as RNAs represent new potential biomarkers and drug targets (16, 17). Currently, a growing number of studies on WT have identified many key mRNAs that are closely associated with the prognosis of this tumor (18, 19). It is well known that inter-individual heterogeneity usually constitutes only a more traditional prognostic system. For example, risk stratification based on the TNM staging system alone is not sufficient, nor is it sufficient to provide an accurate prediction of survival outcomes. A study by Lin et al. (20) identified a 5 mRNA signature as a new potential prognostic biomarker for WT, beyond which models have not been over reported. Therefore, additional prognostic models are needed to predict survival outcomes in pediatric WT patients.

This study was a comprehensive study to analyze differential genes in WT samples through multiple datasets and to develop validated gene signatures for predicting prognosis in WT patients, as well as to further screen key genes to provide a research basis for future biological treatment and clinical diagnosis of WT.

## Methodology

### Data sources

RNA-seq data and clinical information for TARGET-WT were downloaded from the UCSC Xena platform (http://xena.ucsc.edu/), which included 126 cancer tissue samples and six paracancer tissue samples. The TCGA expression matrix was obtained by data fusion and ID transformation of raw TCGA counts data. Searches were performed in Gene Expression Omnibus (GEO, https://www.ncbi.nlm.nih.gov/geo/) using the keyword “Wilms tumor” followed by manual review and selection of cohorts containing miRNA, mRNA expression, GSE57370 (21, 22) (Platform: GPL16770), containing 62 WT cancer tissues and four non-cancerous tissues; GSE73209 (23) (Platform: GPL10558) containing 32 WT cancer tissues and 6 noncancerous tissues. If more than one probe detected the same miRNA expression during the analysis, the average of that miRNA expression was taken as the expression value of that miRNA. For the analysis of patient clinical information, the clinical information of patients with unknown survival times and those equal to zero were deleted.

### Differential Expression Analysis

We applied the limma package of R software (v4.0.3) to perform normalization and base-2 logarithm conversion for the matrix data for each GEO dataset. “Adjusted P value < 0.05 and |logFC| ≥ 1” were defined as the thresholds for differentially expressed gene screening, and overlapping genes were analyzed by Venn plot using the ggplot2 package to plot heat maps and volcano maps, respectively.

### Target Gene Prediction

The target genes of key miRNAs were predicted using the miRDB online database (http://mirdb.org/).

### Functional Enrichment Analysis

Gene ontology (GO) and Kyoto Encyclopedia of Genes and Genomes (KEGG) pathways analyses were performed on genes using the DAVID 6.8 database (https://david.ncifcrf.gov/). Enrichment results with P < 0.05 or FDR < 0.05 were considered significant.

### Kaplan–Meier Survival Analysis

Survival analysis was performed using Survival in the R package. P-values and HR with 95% CI in Kaplan–Meier curves were derived using log-rank tests and univariate Cox proportional hazards regression.

### LASSO Regression Model Construction

The expression matrix integrating the initial genes of the model with patient survival status and survival time was constructed. Furthermore, the LASSO regression algorithm was used for feature selection, and 10-fold cross-validation was used to determine the parameters among which the key genes associated with the patient survival cycle were screened. The genes obtained from the LASSO regression were then subjected to multifactorial Cox regression analysis, and the multifactorial regression coefficients of each gene were calculated to construct a risk score equation. Based on the median risk score, the patients were divided into high-risk and low-risk groups. Kaplan–Meier survival curve analysis was used to compare the overall survival time of the two groups, and the predictive value of the genetic markers was evaluated using time-related ROC.

### Single-Gene Enrichment Analysis (GSEA)

We obtained the GSEA software (version 3.0) from the GSEA website, divided the samples into high and low expression groups based on the median value of gene expression levels, and downloaded the c2.cp.kegg.v7.4.symbols.gmt and h.all.v7.4.symbols.gmt from the Molecular Signatures Database (symbols.gmt subsets) to evaluate relevant pathways and molecular mechanisms based on gene expression profiles and phenotypic groupings, setting a minimum gene set of five and a maximum gene set of 5000, with 1000 resamplings and a screening condition of FDR < 0.25 and P < 0.05.

### Statistical Analysis

R software (v4.0.3) was used for data analysis, and the Wilcoxon rank sum test was used between gene and miRNA expression groups in the data samples. Cox regression analysis was performed using SPSS 25.0, and P < 0.05 was considered statistically significant.

## Results

### Screening of Differentially Expressed Genes in Wilms Tumor

**Figure 1:**
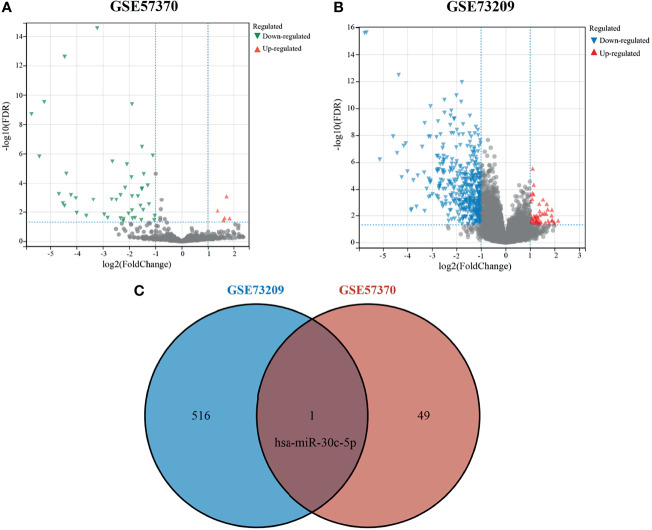
Differentially expressed genes in Wilms tumor. **(A)** Volcano plot showing differentially expressed miRNAs in GSE57370; **(B)** Volcano plot showing differentially expressed genes in GSE73209; **(C)** Venn plot showing intersecting genes.

The GEO database was used to obtain the WT-related miRNA expression dataset GSE57370, which included 62 WT tissue samples and four normal kidney tissue samples, and the mRNA expression dataset GSE73209, which included 32 WT tissue samples and six normal kidney tissue samples. Using |logFC|≥1 and adjusted P < 0.05 as screening thresholds, five upregulated miRNAs and 45 downregulated miRNAs were obtained in GSE57370 (**Figure 1A**), and a total of 58 upregulated genes and 459 downregulated genes were obtained in GSE73209 (**Figure 1B**). Lastly, one intersection gene, hsa-miR-30c-5p (**Figure 1C**), was obtained for both sets of differentially expressed genes.

### Target Gene Prediction of hsa-miR-30c-5p

**Figure 2:**
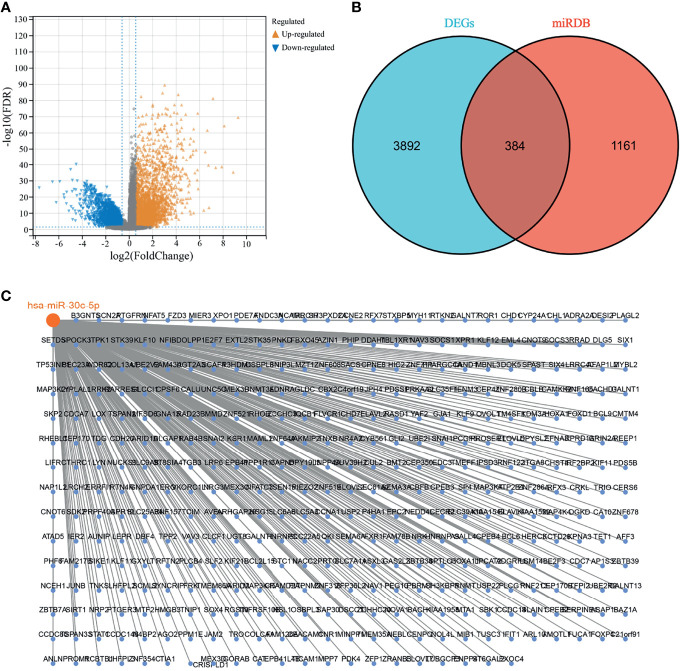
hsa-miR-30c-5p target genes. **(A)** Wilms tumor differentially expressed genes in the Target database; **(B)** Venn diagram showing the intersection of differentially expressed genes and target genes; **(C)** hsa-miR-30c-5p with its target genes.

RNA-seq data of TARGET-WT were downloaded from the UCSC Xena platform (http://xena.ucsc.edu/), in which a total of 126 cancer tissue samples and six para-carcinoma tissue samples were obtained, with |logFC|≥1 and adjusted P < 0.05 as screening thresholds, and 2217 and 2059 upregulated and downregulated genes, respectively (**Figure 2A**). The miRDB database was used to predict the target genes for hsa-miR-30c-5p, and 1545 target genes were obtained. The differentially expressed genes and target genes overlapped separately, and 384 genes were obtained (**Figure 2B**). Lastly, hsa-miR-30c-5p was related to 384 genes, as shown in **Figure 2C**.

### Functional Enrichment Analysis of 384 Genes

**Figure 3:**
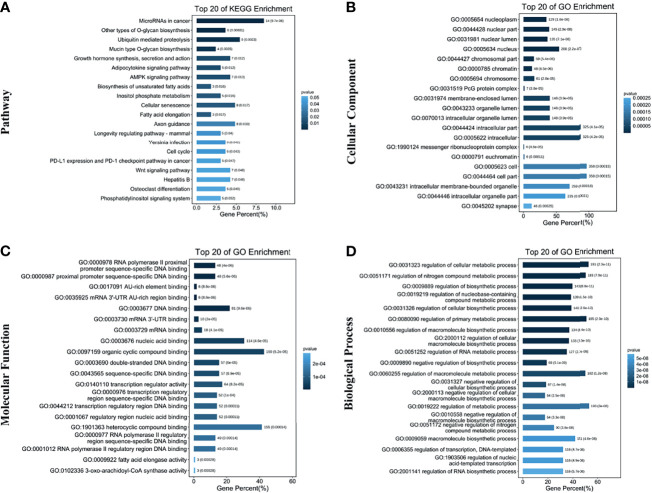
Functional enrichment analysis of target genes. **(A)** Top 20 terms in the KEGG pathway enrichment; **(B)** Top 20 terms in the cellular component enrichment; **(C)** Top 20 terms in the molecular function enrichment; **(D)** Top 20 terms in the biological process enrichment.

Subsequently, 384 genes were analyzed for the KEGG pathway and GO functional enrichment. KEGG involved a total of 20 pathways, mainly enriched in microRNAs in cancer, other types of O-glycan biosynthesis, and Ubiquitin mediated proteolysis (**Figure 3A**). GO enrichment analysis showed that 384 genes were mainly enriched in the nucleoplasm, regulation of the cellular metabolic process, etc. (**Figures 3B–D**).

### Kaplan–Meier Survival Analysis of 384 Genes

Kaplan–Meier survival analysis of 384 genes was used to analyze the relationship between gene expression and the overall survival of WTs. The results showed that 25 genes were significantly associated with the overall survival of Wilms tumor, namely ADRA2A, BCL6, CA10, CCNA1, CTHRC1, DGKD, EPB41L4B, ERRFI1, GALNT3, JAM2, LRRC40, MTF2, NCEH1 NEBL, OSBPL3, PDS5B, PDSS1, RFX3, ROR1, RTKN2, SLAIN1, SPTLC3, TICAM1, TUBGCP3, and ZFP36L2 (see **Table 1**).

**Table 1:** The 25 genes related to overall survival according to Kaplan–Meier survival analysis.

Gene	P-value	HR	95% CI
ADRA2A	0.028	0.544	0.315-0.937
BCL6	0.002	0.409	0.233-0.719
CA10	0.004	0.445	0.255-0.778
CCNA1	0.017	1.951	1.126-3.380
CTHRC1	0.011	0.489	0.282-0.848
DGKD	0.048	1.731	1.004-2.984
EPB41L4B	0.018	0.519	0.302-0.892
ERRFI1	0.016	1.961	1.132-3.398
GALNT3	0.020	0.521	0.301-0.903
JAM2	0.044	0.572	0.332-0.986
LRRC40	0.046	1.732	1.009-2.973
MTF2	0.029	1.833	1.063-3.161
NCEH1	0.047	0.580	0.339-0.993
NEBL	0.047	0.578	0.336-0.992
OSBPL3	0.008	0.472	0.272-0.818
PDS5B	0.025	1.863	1.080-3.215
PDSS1	0.044	1.743	1.016-2.991
RFX3	0.031	1.832	1.057-3.174
ROR1	0.042	0.571	0.332-0.980
RTKN2	0.025	1.864	1.081-3.213
SLAIN1	0.040	1.759	1.025-3.018
SPTLC3	0.007	0.468	0.268-0.816
TICAM1	0.019	0.521	0.302-0.898
TUBGCP3	0.026	1.856	1.076-3.200
ZFP36L2	0.016	0.510	0.294-0.884

### Construction of a Prognostic Risk Model

**Figure 4:**
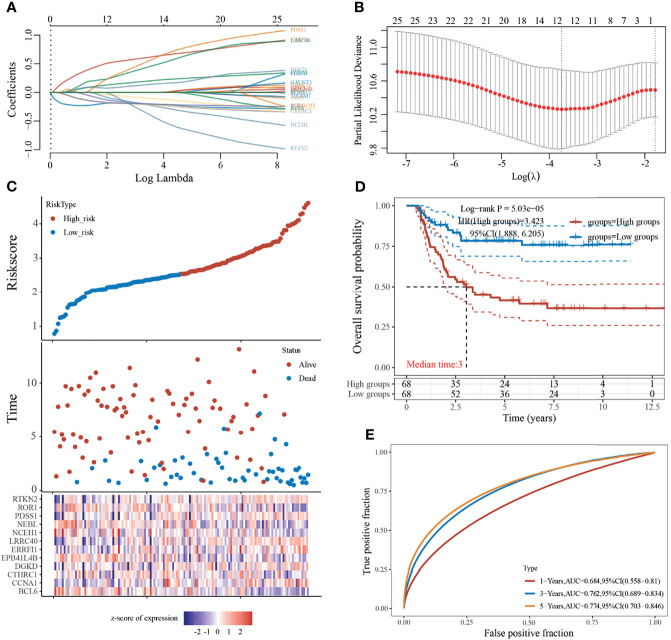
Construction of prognostic risk model. **(A)** Coefficients of selected features are shown by lambda parameter; **(B)** Partial likelihood deviance versus log (λ) was drawn using the LASSO Cox regression model; **(C)** Risk score, survival time, and survival status in dataset; **(D)** Kaplan–Meier survival analysis of the gene signature; **(E)** Time-dependent ROC analysis of the gene signature.

The LASSO regression model screened 25 genes to identify key genes affecting WT prognosis, and the model was optimal when the number of genetic variables included in the model was 12 (lambda.min = 0.0238, **Figures 4A, B**), which were the key genes associated with the prognosis of WT patients, namely BCL6, CCNA1, CTHRC1, DGKD EPB41L4B, ERRFI1, LRRC40, NCEH1, NEBL, PDSS1, ROR1, and RTKN2. Moreover, a prediction model based on the 12 gene signatures was constructed (**Figure 4C**) whose predicted risk scores consisted mainly of the following:


$$\begin{array}{c} \text{Risk score}=(-0.2011)*\text{BCL}6+(0.1837)*\text{CCNA}1+(-0.1889)*\text{CTHRC}1+(0.1227)*\text{DGKD}\\ +(-0.0891)*\text{EPB}41\text{L}4\text{B}+(0.5903)*\text{ERRFI}1+(0.3992)*\text{LRRC}40+(-0.1926)*\text{NCEH}1\;\\ +(-0.\;2065)*\text{NEBL}+(0.4799)*\text{PDSS}1+(-0.1969)*\text{ROR}1*(-0.2957)*\text{RTKN}2 \end{array}$$

Risk scores were calculated according to the formula, and the median risk score was used as the threshold to divide the sample into high-risk and low-risk groups. The results for the Kaplan–Meier survival analysis showed that patients in the high-risk group had a significantly worse prognosis than those in the low-risk group (**Figure 4D**). In addition, the accuracy of the model in predicting patients’ OS period was verified using subject working curves, and we found that the risk model predicted AUC values of 0.684, 0.762, and 0.774 for the prognosis of WT patients at 1, 3, and 5 years, respectively. These results indicate that the model has some accuracy in predicting the prognosis survival of WT patients (**Figure 4E**).

### Univariate and Multifactor Cox Regression Analysis of Risk Score Grouping and Clinicopathological Indicators

**Figure 5:**
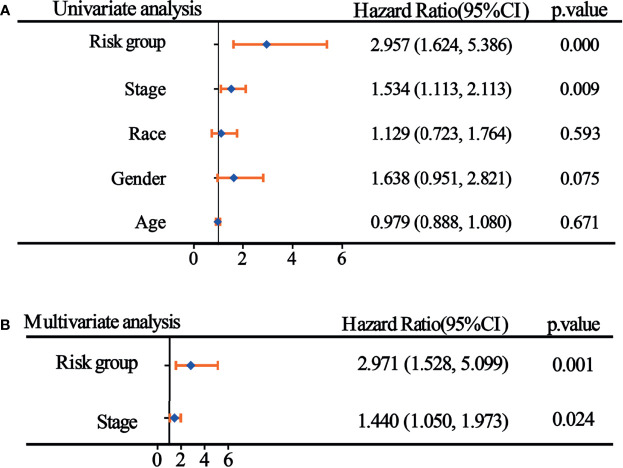
Univariate and multifactor Cox regression analyses of risk score groupings and clinicopathological indicators. **(A)** Results of single-factor Cox analysis; **(B)** Results of multifactor Cox regression analysis.

Single-factor Cox analysis was used to analyze the relationship between clinicopathological indicators and prognosis, and the results demonstrated that risk score grouping and tumor stage were significantly associated with patient prognosis (**Figure 5A**). To adjust the interaction between variables and to understand the independent prognostic value of variables, indicators with significant single-factor analysis were introduced into the model for multifactor regression analysis, and the results of Jie showed that risk score grouping and tumor stage could be used as independent predictors of patient prognosis for WT (**Figure 5B**).

### Single-Factor and Multifactor Cox Analysis for 12 Genes

Univariate and multifactor Cox analyses were performed for the 12 genes in the model. The results of the univariate analysis showed that BCL6, CCNA1, EPB41L4B, ERRFI1, LRRC40, NEBL, and ROR1 were significantly associated with patient prognosis (**Table 2**). Consequently, they were included in the multifactor analysis, and the results showed that ERRFI1 and ROR1 could be used as independent predictors of patient prognosis (**Table 3**).

**Table 2:** Univariate cox analysis of 12 genes.

Gene	B	SE	P-value	HR 95%CI
BCL6	-0.489	0.158	0.002	0.613 (0.450-0.835)
CCNA1	0.227	0.109	0.037	1.255 (1.013-1.555)
CTHRC1	-0.091	0.088	0.297	0.913 (0.769-1.084)
DGKD	0.360	0.191	0.059	1.434 (0.986-2.085)
EPB41L4B	-0.361	0.183	0.049	0.697 (0.487-0.998)
ERRFI1	0.378	0.161	0.019	1.459 (1.064-2.001)
LRRC40	0.624	0.264	0.018	1.866 (1.112-3.133)
NCEH1	-0.405	0.255	0.112	0.667 (0.404-1.099)
NEBL	-0.275	0.113	0.015	0.760 (0.609-0.948)
PDSS1	0.364	0.276	0.188	1.439 (0.837-2.471)
ROR1	-0.246	0.109	0.025	0.782 (0.631-0.969)
RTKN2	0.216	0.178	0.225	1.240 (0.876-1.758)

**Table 3:** Multivariate cox analysis of genes.

Gene	B	SE	P-value	HR 95%CI
BCL6	-0.284	0.194	0.144	0.753 (0.515-1.102)
CCNA1	0.207	0.129	0.109	1.230 (0.955-1.585)
EPB41L4B	-0.005	0.230	0.983	0.995 (0.634-1.561)
ERRFI1	0.620	0.183	0.001	1.858 (1.298-2.660)
LRRC40	0.375	0.329	0.254	1.455 (0.763-2.773)
NEBL	-0.235	0.140	0.094	0.790 (0.600-1.041)
ROR1	-0.249	0.126	0.048	0.780 (0.609-0.998)

### Expression of ERRFI1 and ROR1 and Prognosis

**Figure 6:**
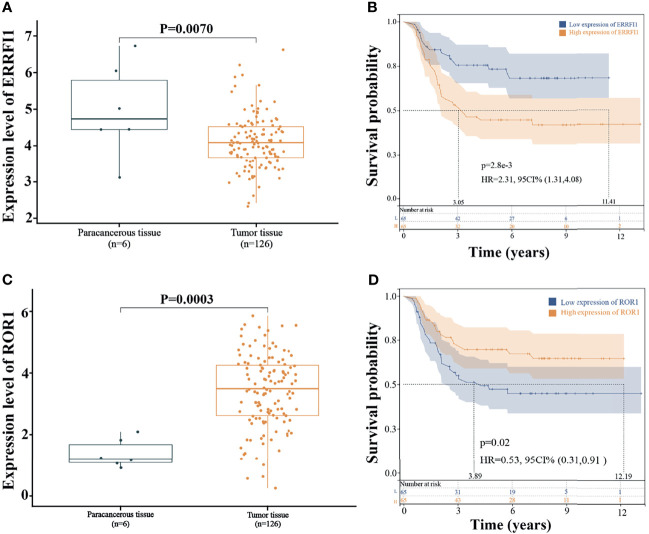
Expression and prognosis of ERRFI1 and ROR1. **(A)** Expression level of ERRFI1 in the TARGET-WT database; **(B)** Prognosis analysis of ERRFI1 in the TARGET-WT database; **(C)** Expression level of ROR1 in the TARGET-WT database; **(D)** Prognosis analysis of ROR1 in the TARGET-WT database.

The expression levels of ERRFI1 and ROR1 were analyzed by integrating 126 cases of WT tissue samples and six cases of paracancerous tissue samples from the TARGET database. The results showed that ERRFI1 was significantly downregulated in the WT (**Figure 6A**), and ROR1 was significantly upregulated (**Figure 6C**). KM survival analysis showed that high expression of ERRFI1 and low expression of ROR1 were significantly associated with poor patient prognosis (**Figures 6B, D**).

### Single-Gene Functional Enrichment Analysis

**Figure 7:**
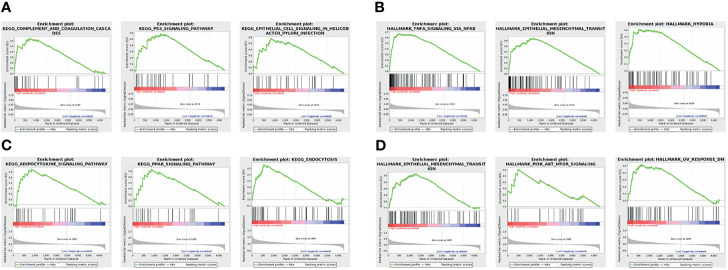
Single-gene functional enrichment analysis. **(A)** Results of ERRFI1 enrichment analysis in the KEGG pathway; **(B)** Results of ERRFI1 enrichment analysis in the HALLMARK pathway; **(C)** Results of ROR1 enrichment analysis in the KEGG pathway; **(D)** Results of ROR1 enrichment analysis in the HALLMARK pathway.

The GSEA results showed that the three KEGG pathways and the HALLMARK pathway were most significantly associated with ERRFI1 high expression. Among them, high ERRFI1 expression was mainly enriched in the complement and coagulation cascade, the p53 signaling pathway, and epithelial cell signaling in *H. pylori* infection-related cells (**Figure 7A**) High expression of ERRFI1 was positive for TNF-α signaling pathway *via* NK-κB, epithelial-mesenchymal transition, and hypoxia (**Figure 7B**) signaling pathway, PPAR signaling pathway, and endocytosis (**Figure 7C**). High ROR1 expression was positive for epithelial-mesenchymal transition, PI3K-AKT-mTOR signaling, and UV response (**Figure 7D**).

## Discussion

Numerous studies have reported that mRNA plays a crucial role in the tumorigenesis development of WT (24). However, with the development of detection techniques, a single mRNA expression pattern is no longer sufficient to accurately predict the prognosis of WT. In addition, the role of miRNAs in altered gene expression should not be neglected. Zhu et al. (25) reported that miR-92a-3p inhibited the proliferation, migration, and invasion of WT cells by regulating the NOTCH1 signaling pathway. Therefore, the identification of differentially expressed miRNAs represents a promising strategy. However, heterogeneous results are primarily generated by studies with relatively limited sample sizes, several candidate miRNAs, or a lack of experimental validation. In this study, we identified one hub miRNA—miR-30c-5p—by integrating the dataset and constructed a 12-gene marker-based prognostic prediction model for WT based on the prediction of miR-30c-5p target genes and the results of WT differentially expressed gene analysis.

MiR-30 c-5p (Previous ID: miR-30c) was first identified in 2002 by Lagos-Quintana et al. (26) from mouse heart and brain tissues, and its sequence is highly conserved across species. Several studies have reported that miR-30c-5p is aberrantly expressed in different tumor tissues, sera, and cell lines and is associated with clinical features and prognostic factors in a variety of cancers, including lung cancer (27), breast cancer (28), and colorectal cancer (29). In 2010, Heinzelmann et al. (30) screened 12 miRNAs, including miR-30c-5p, which were lowly expressed in highly invasive renal clear cell carcinoma with early metastasis. In an in-depth study, it was found that miR-30c-5p expression levels were significantly lower in primary tumors with metastasis compared to normal kidney tissue and primary renal clear cell carcinoma without metastasis, and miR-30c-5p expression levels were significantly correlated with the 5-year progression-free survival of patients (31). Thus, miR-30c-5p may be a useful indicator for the early prediction of renal cancer metastasis, and different expression levels may be associated with specific distant metastasis. This study focused on the role of the target genes of miR-30c-5p in WT. We predicted the target genes of miR-30c-5p and obtained a total of 384 genes in combination with differentially expressed genes in TARGET-WT. Furthermore, KM survival analysis of these 384 genes was performed to obtain 25 genes associated with cervical cancer prognosis, and LASSO Cox regression was used as a machine-learning algorithm to construct a prognostic risk model with 12 gene signatures (i.e., BCL6, CCNA1, CTHRC1, DGKD, EPB41L4B, ERRFI1, LRRC40, NCEH1, NEBL, PDSS1, ROR1, and RTKN2), and the patients were divided into high-risk and low-risk groups according to risk scores. The prognosis of patients in the high-risk group was significantly worse than that in the low-risk group, and the working curves of subjects were used to verify that the model had good predictive ability for 1, 3, and 5-year prognoses of WT patients, and the risk group could be used as an independent predictor of WT prognosis in patients. Previously, gene and miRNA signatures were developed for WT prognosis prediction. However, the number of models is far from enough.

Finally, we performed univariate and multivariate Cox analyses for each of the 12 genes in the model and found that ERRFI1 and ROR1 served as independent predictors. ERBB receptor Feedback Inhibitor 1 (ERRFI1), the product of mitogen-inducible gene 6, through its ERBB-binding region, docks with the EGFR kinase structural domain docking, inhibiting EGFR activation and downstream signaling (32). We found low expression of ERRFI1 in WT cancer tissues after comparing its expression in WT, and it has been reported that ERRFI1 is also frequently mutated or downregulated in breast cancer (33), lung cancer (34), and glioblastoma (35). However, we found that high ERRFI1 expression was significantly associated with a poor prognosis in WT patients.

ERRFI1 is considered a tumor suppressor that directly inhibits epidermal growth factor receptors. However, new studies have found that the role of ERRFI1 depends on EGFR levels; therefore, in a low EGFR setting, downregulation of ERRFI1 leads to higher migration rates and promotes cell growth (36). In the Jäger K et al. (37) human study, ERRFI1 upregulation was found to be significantly associated with poor prognosis in metastatic melanoma, and in the present study, ERRFI1 upregulation was similarly found to be associated with poor prognosis in WT. Receptor tyrosine kinase-like orphan receptor 1 (ROR1) is a member of the ROR family of type I transmembrane receptors with ligand-bound extracellular and intracellular tyrosine kinase domains.

During embryogenesis, ROR1 plays a physiological role in neural, auditory, skeletal, and vascular organogenesis, but studies have shown that ROR1 is absent or expressed at low levels in most adult tissues (38, 39). However, as an oncoprotein, ROR1 can reemerge in hematological and solid tumors, especially in histologically advanced tumors, where ROR1 may promote tumor cell migration through Wnt5a signaling or interaction with other receptors (40, 41). In the present study, we found high expression of ROR1 in WT cancer tissues. Hodjattallah Rabbani et al. (42) verified that detecting a high level of ROR1 expression in blood cells may help in the early detection of renal malignancies. Notably, Kaplan–Meier survival analysis found that low ROR1 expression was significantly associated with poor patient prognosis, a result that is contrary to most studies that have partially demonstrated poor prognosis in patients with high ROR1 expression, such as ovarian cancer (43), colorectal cancer (44), and so on. Therefore, the prognostic predictive role of ROR1 expression in WT remains to be explored in depth.

In summary, this study constructed a 12-gene signature prognostic risk model based on the target genes of miR-30c-5p and determined that ERRFI1 and ROR1 could be used as independent predictors of WT prognosis in patients. However, this study has several shortcomings. These include the lack of biological behavior studies for the identified ERRFI1 and ROR1 and the fact that there are few studies on ERRFI1 and ROR1 in WT. However, it also has potential research value. In addition, WT is a relatively rare primary malignancy, and more clinical samples and survival information are needed to validate the results of this study. Although the risk model constructed in this study displayed better performance, the LASSO Cox algorithm used will adjust the parameters appropriately during the calculation to avoid degradation of the algorithm’s performance (45). Therefore, more accurate machine-learning methods, a larger number of clinical samples, and *in vitro* experiments need to be further developed in future studies.

## Data Availability Statement

The original contributions presented in the study are included in the article/supplementary material. Further inquiries can be directed to the corresponding author.

## Author Contributions

All authors contributed to the article and approved the submitted version.

## Conflict of Interest

The authors declare that the research was conducted in the absence of any commercial or financial relationships that could be construed as a potential conflict of interest.

## Publisher’s Note

All claims expressed in this article are solely those of the authors and do not necessarily represent those of their affiliated organizations, or those of the publisher, the editors and the reviewers. Any product that may be evaluated in this article, or claim that may be made by its manufacturer, is not guaranteed or endorsed by the publisher.
